# Serological evidence of West Nile virus infection among birds and horses in some geographical locations of Iran

**DOI:** 10.1002/vms3.342

**Published:** 2020-08-28

**Authors:** Hasan Bakhshi, Cécile Beck, Sylvie Lecollinet, Maëlle Monier, Laurence Mousson, Sedigheh Zakeri, Abbasali Raz, Kourosh Arzamani, Leila Nourani, Navid Dinparast‐Djadid, Anna‐Bella Failloux

**Affiliations:** ^1^ Malaria and Vector Research Group Biotechnology Research Center Pasteur Institute of Iran Tehran Iran; ^2^ EURL on Equine Diseases ANSES Animal Health Laboratory UMR 1161 Virology ANSES INRAE ENVA Maisons‐Alfort France; ^3^ Department of virology, Arboviruses and Insect Vectors Institut Pasteur Paris France; ^4^ Vector‐borne Diseases Research Center North Khorasan University of Medical Sciences Bojnurd Iran

**Keywords:** ELISA, Iran, Usutu virus, vertebrate hosts, West Nile virus

## Abstract

Recent expansion of arboviruses such as West Nile (WNV), Usutu (USUV), and tick‐borne encephalitis (TBEV) over their natural range of distribution needs strengthening their surveillance. As common viral vertebrate hosts, birds and horses deserve special attention with routine serological surveillance. Here, we estimated the seroprevalence of WNV, USUV and TBEV in 160 migrating/resident birds and 60 horses sampled in Mazandaran, Golestan, North Khorasan, Kordestan provinces and Golestan province of Iran respectively. ELISA results showed that of 220 collected samples, 32 samples (14.54%), including 22 birds and 10 horses, were positive. Microsphere immunoassay results showed that 16.7% (10/60) of horse blood samples collected in Golestan province were seropositive against WNV (7; 11.7%), *Flavivirus* (2; 3.3%) and seropositive for USUV or WNV (1; 1.7%). Furthermore, micro virus neutralization tests revealed that four of seven ELISA‐positive bird blood samples were seropositive against WNV: two Egyptian vultures, and one long‐legged buzzard collected in Golestan province as well as a golden eagle collected in North Khorasan province. No evidence of seropositivity with TBEV was observed in collected samples. We showed that WNV, responsible for neuroinvasive infection in vertebrates, is circulating among birds and horses in Iran, recommending a sustained surveillance of viral infections in animals, and anticipating future infections in humans.

## Graphical abstract

We estimated the seroprevalence of WNV, USUV and TBEV in 160 birds and 60 horses. ELISA results showed that 32 samples were positive: Microsphere immunoassay results showed that 16.7% of horse blood samples were seropositive against WNV, Flavivirus and seropositive for USUV or WNV. Furthermore, micro virus neutralization tests revealed that four of seven ELISA‐positive bird blood samples were seropositive against WNV.

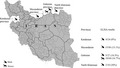

## 1 INTRODUCTION

Mosquito‐borne arboviruses are responsible for millions of human cases and thousands of deaths each year (Caraballo & King, 2014). Mosquito‐borne arboviruses are commonly reported in Iran (Shahhosseini et al., 2017) beside tick‐borne (Telmadarraiy, Chinikar, Vatandoost, Faghihi, & Hosseini‐Chegeni, 2015) and sandfly‐borne arboviruses (Shiraly, Khosravi, & Farahangiz, 2017). Serological studies have confirmed the circulation of these viruses in vertebrate hosts in Iran (Aghaie et al., 2014; Chinikar, Shah‐Hosseini, Mostafavi, Moradi, Khakifirouz, Jalali, & Fooks, 2013a; Chinikar, Shah‐Hosseini, Mostafavi, Moradi, Khakifirouz, Jalali, Goya, et al., 2013b; Shiraly et al., 2013b, 2013a, 2017; Ziyaeyan et al., 2018). As Iran shares borders with countries where these viruses cause epidemics, the country becomes at risk for a virus introduction in an environment where mosquito vectors are well established. The recent establishment of the mosquitoes *Aedes albopictus* (Doosti et al., 2016), and *Aedes unilineatus* (Yaghoobi‐Ershadi et al., 2017) poses the threat of emergence of associated arboviruses including chikungunya (CHIKV; *Alphavirus*, Togaviridae) from Pakistan (Rauf, Manzoor, Mehmood, & Bhatti, 2017; Sahibzada, Khurshid, Khan, Zafar, & Siddiqi, 2018) and Iraq (Barakat et al., 2016) as well as Rift Valley fever (RVFV; *Phlebovirus*, Phenuiviridae) (Chinikar, Shah‐Hosseini, Mostafavi, Moradi, Khakifirouz, Jalali, & Fooks, 2013a) in south‐eastern region of Iran. West Nile virus (WNV, Genus *Flavivirus*, Family Flaviviridae) is responsible for severe neuroinvasive infections in vertebrates. This virus is the most prevalent *Culex*‐transmitted virus, frequently reported in Iran (Naficy & Saidi, 1970; Saidi, Tesh, Javadian, & Nadim, 1976): Many studies have found the infection of humans (Sharifi, Mahmoudian, & Talebian, 2010), horses (Ahmadnejad et al., 2011) and migratory birds (Fereidouni et al., 2011) in Iran. To our knowledge, there is no report of infection of vectors with Zika virus (ZIKV; *Flavivirus*, Flaviviridae), yellow fever virus (YFV; *Flavivirus*, Flaviviridae), Usutu virus (USUV; *Flavivirus*, Flaviviridae) and CHIKV in the country.

For most of these arboviruses, there is no efficient vaccine alternative, and early detection of virus circulation becomes an alert system for implementing vector control measures. In this study, we determined the seroprevalence of IgG antibodies against WNV, USUV and tick‐borne encephalitis virus (TBEV) in migratory/resident birds, and horses in four provinces in Iran, including Mazandaran, Golestan, North Khorasan and Kordestan.

## 2 MATERIALS AND METHODS

### 2.1 Ethics statements

The animal procedures were approved by the committee of animal ethics of Pasteur Institut of Iran (IR.PII.REC.1398.012).

### 2.2 Study area and Sample collection

**FIGURE 1:**
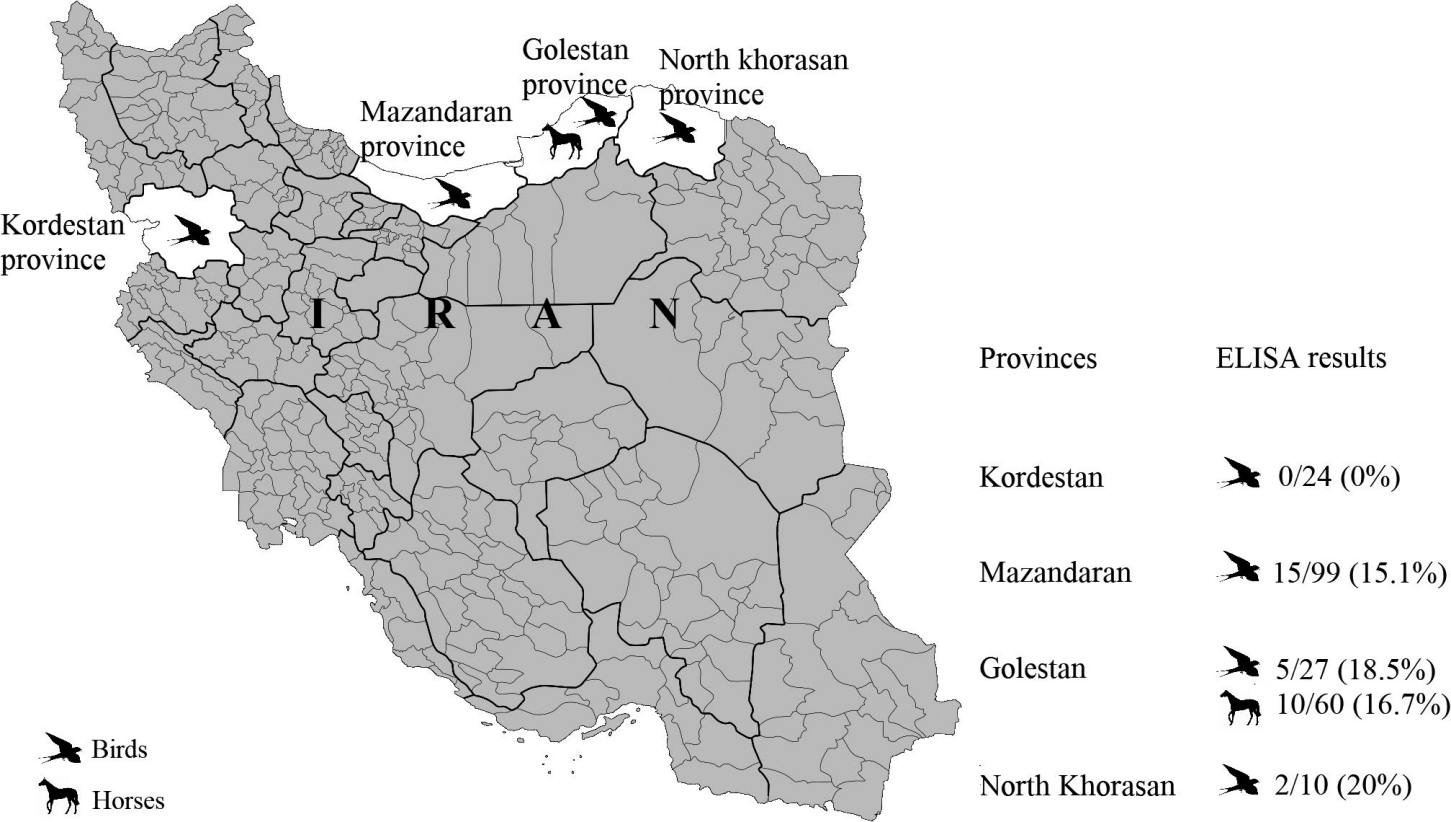
The study area in Golestan, North khorasan and Mazandaran provinces located in north, and Kordestan province located in west of Iran. Bird blood samples were collected in four provinces, and horse blood samples were collected in Golestan province. The map was built using the open source map site “https://commons.wikimedia.org/wiki/Atlas_of_the_world”

The serum samples of 160 birds were collected in four provinces, located in north and west of Iran, where migratory/resident birds are frequently found (Figure 1). In addition, a total of 60 healthy horses were randomly selected in Gonbad‐Kavus County (Golestan province), where horse breeding centres are located, and blood sampling was carried out (Table S1). The bird blood samples were collected in some counties of four provinces: Mazandaran (Amol, Amol County: 13; Ezbaran, Fereydunkenar County: 25; Fereydunkenar, Fereydunkenar County: 21; Sorkhrud, Mahmudabad County: 3; Babol, Babol County: 33; Behshahr, Behshahr County: 2; Sari, Sari county: 1; Savadkouh, Savadkouh, county: 1), Kordestan (Sanandaj, Sanandaj County: 24), Golestan (Genareh, Gorgan County: 27) and North Khorasan Province (Bojnurd, Bojnurd County: 10). Collection of horse and bird blood samples was carried out in 2017 and 2018 respectively; after identification of a total number of 220 samples, belonging to 30 species (10 families, Table S2), they were serologically examined.

### 2.3 Serological surveillance of birds and horses

#### 2.3.1 ELISA

The serological diagnosis was made using a cELISA test (ID Screen WNV Competition ELISA Kit, ID Vet, France) on birds and horse sera samples. Interpretation was performed according to the manufacturer's instructions. The threshold value for considering a serum as positive was % OD sample/negative control (%S/N) ≤40% as recommended by the manufacturer. Those with a 40% <%S/N ≤ 50% or less and greater than 40% were considered doubtful, and those with a %S/N > 50% were considered negative. This test has been used to give an indication of the presence or absence of anti‐WNV antibodies in sera, but cross‐reactions with other Flaviviruses may occur.

#### 2.3.2 Microsphere immunoassay

A *Flavivirus* xMAP microsphere immunoassays (MIAs) was performed on the 10 horse blood samples positive with the ID Screen WNV competition ELISA (Table S3). Purified recombinant envelope domain III (rEDIII) proteins of WNV, USUV and TBEV were used for the capture of specific IgG antibodies. A recombinant soluble ectodomain of WNV envelope (E) glycoprotein (WNV.sE) which identifies infection by every Flaviviruses in the same way as the competitive ELISA test and WNV.EDIII, USUV.EDIII and TBEV.EDIII were coupled to MagPlex^®^ Microspheres using the Bio‐Plex Amine coupling kit (Bio‐Rad Laboratories, Hercules, CA, USA) according to the manufacturer's instructions, as described in Beck et al. (2015). Five micrograms of WNV.sE or TBEV.EDIII or 50 µg of WNV.EDIII and USUV.EDIII were coupled with the beads. The sera (diluted 1/100) were then tested with the MIA technology using an equimolar mixture of the four beads as previously described in Beck et al. (2015). The secondary biotinylated goat anti‐horse IgG (Jackson immunoresearch) antibody diluted at 1:500 was then added and the Antigen/Antibody complex was revealed by streptavidin R‐phycoerythrin conjugate (SAPE). For each antigen, the diagnostic cut off was determined from the mean of median of fluorescence (MFI) values of 66 horse‐negative sera plus 3 standard deviations of the mean. The 66 sera used to determine the cut off were sampled in Poland (35 sera) and Ireland (31 sera) and were all found negative with the ID screen West Nile competition kit (IDVet). In cases of positive reactions with several rEDIIIs for viruses belonging to the Japanese serocomplex (ie USUV and WNV) an animal was considered infected with a specific *Flavivirus* if the corresponding bead coupled to rEDIII generated an MFI at least two‐fold greater than that generated with the other beads. If a twofold difference could not be achieved, the sample was considered to be infected with WNV or USUV. The sample was considered positive for an undetermined *Flavivirus* if it reacted with WNV.sE but not with any of the rEDIIIs.

#### 2.3.3 Micro virus neutralization tests

Of 22 ELISA‐positive bird sera (Table S3), seven sera had enough volume to be tested by microneutralization tests (MNTs). These samples were investigated through MNT against WNV, USUV and TBEV for the detection of specific neutralizing antibodies against WNV (strain Is98, Genbank ID AF481864.1, provided by P. Desprès, IPP), USUV (strain France 2018) and TBEV (strain Hypr, Genbank ID U39292.1) following the protocol described in Beck et al. (2015). Fifteen sera were untested by MNT due to insufficient volume.

A serum was considered positive if cells were protected at the 1:10 serum dilution for WNV and USUV and the 1:20 serum dilution for TBEV. Owing cross‐neutralization between Flaviviruses especially in the same serocomplex, we identified the infecting *Flavivirus* by considering the virus with the highest neutralization capacity, and with neutralization titres that differ by at least a fourfold factor.

## 3 RESULTS

ELISA results showed that of 220 collected samples, 32 samples (14.54%), including 22 birds and 10 horses, were positive (positive: %S/N ≤ 40%; doubtful: 40% <S/N ≤ 50%; negative: S/N > 50%) (Table S3). Of 22 bird blood samples tested, no positive sample was found in Kordestan province. However, we found that 15.1% (15/99), 18.5% (5/27) and 20% (2/10) of bird samples were positive in Mazandaran, Golestan and North Khorasan provinces respectively (Figure 1). Microsphere immunoassay results showed that 16.7% (10/60; 95% CI [15.4, 17.9]) of horse blood samples at an age between 1.5 and 6 years (Table 1; Table S1), collected in Golestan province, were seropositive against WNV (7; 11.7%; 95% CI [10.4, 12.9]), *Flavivirus* (2; 3.3%; 95% CI [2.1, 4.6]) and seropositive for USUV or WNV (1; 1.7%; 95% CI [0.4, 2.9]). The percentage was based on total sample numbers, assuming all cELISA negative would also be negative by MIAs. Furthermore, micro virus neutralization test revealed that four of seven tested ELISA‐positive bird samples were seropositive against WNV (table S4): two Egyptian vultures (*Neophron percnopterus*) and one long‐legged buzzard (*Buteo rufinus*), collected in Golestan province as well as a golden eagle (*Aquila chrysaetos*) collected in North Khorasan province (Table 1, Table S2). No evidence of TBEV infection was detected in the sampled animals.

**TABLE 1:** Seroprevalence of IgG against WNV, USUV and TBEV in study areas. A total of 10 horse blood samples collected in Golestan province were seropositive against WNV (7), *Flavivirus* (2) and WNV or USUV (1). Of seven ELISA‐positive samples, four bird blood samples collected in North Khorasan and Golestan provinces were seropositive against WNV

Place of collection	Species	Gender	Age	Seropositive for
Birds				
Genareh, Golestan	*Neophron percnopterus*	NA	NA	WNV
	*Buteo rufinus*	NA	NA	WNV
	*Neophron percnopterus*	NA	NA	WNV
Bojnurd, North Khorasan	*Aquila chrysaetos*	NA	NA	WNV
Horses				
Gonbad‐Kavus, Golestan	*Equus ferus caballus*	M	2	WNV
	*Equus ferus caballus*	F	2	WNV
	*Equus ferus caballus*	M	2	WNV
	*Equus ferus caballus*	F	2	WNV
	*Equus ferus caballus*	M	5	*Flavivirus*
	*Equus ferus caballus*	F	1.5	*Flavivirus*
	*Equus ferus caballus*	M	2	WNV
	*Equus ferus caballus*	M	2	WNV
	*Equus ferus caballus*	M	6	WNV
	*Equus ferus caballus*	F	2.5	USUV or WNV

## 4 DISCUSSION

Our study indicates that WNV circulates in north of Iran based on positive serologies detected in 220 bird and horse blood samples. We did not find any positive sample in Kordestan province, located in west of the country. WNV has a complex cycle involving primary bird species hosts, primary mosquito vectors (Genus: *Culex*) and humans, horses or rodents as incidental hosts (Vázquez et al., 2011). We also showed that a horse was serologically positive for WNV or USUV. Birds play an important role in introducing WNV (Rappole & Hubalek, 2003) and USUV (Ayadi et al., 2017). Therefore, monitoring migratory/resident birds is a tool to detect any attempts of virus entry. Moreover, serosurvey of infections in horses will also help in making decisions for implementing sanitary measures (Michel et al., 2019).

WNV is considered the most prevalent *Flavivirus* frequently reported in Iran; this virus was detected in mosquitoes in the northwest (Bagheri et al., 2015), north (Shahhosseini et al., 2017) and south (Ziyaeyan et al., 2018) of the country. Positive serologies against WNV were also reported in humans (Sharifi et al., 2010) and horses (Ahmadnejad et al., 2011). With a 13.3% of seroprevalence (10/60) in Golestan province, our results are in concordance with a large‐scale serosurvey on horses implemented in 27 provinces for WNV (Ahmadnejad et al., 2011); they showed that the overall seroprevalence rate was 23.7% in 2008–2009 and in Golestan province, between 1% and 10%. Besides humans and horses, migratory birds play a critical role in introducing WNV in Iran. We found that WNV‐positive birds, including Egyptian vulture, long‐legged buzzard and golden eagle, belonged to Accipitridae family. A larger‐scale survey showed that 15% of sampled birds, collected in 6 provinces of Iran, were serologically WNV positive and among them, 54% concerned common coots (Fereidouni et al., 2011), suggesting that greater the sampling effort is, higher the chance of detecting positive serologies could be.

USUV is closely related to WNV (Saiz & Blazquez, 2017). In humans, Usutu viral RNA has been detected in patients with neurological disorders (Clé et al., 2019; Grottola et al., 2017; Vilibic‐Cavlek et al., 2014). USUV has been also detected in mosquitoes, birds or horses in many African (Nikolay, Diallo, Boye, & Sall, 2011) and European countries (Ashraf et al., 2015). However, there is no report of the presence of this virus in Iran and neighbouring countries. We showed that one horse blood sample was serologically positive for WNV or USUV meaning that USUV may circulate in Iran. This horse was born and always had been in Iran, with no record of leaving the country.

The transmission dynamics of Flaviviruses are mainly influenced by environmental and biological factors. Furthermore, USUV shares some features with WNV. Both USUV and WNV are known as *Culex*‐transmitted mosquitoes, with migratory birds acting as amplifying hosts. Therefore, it is not surprising that in places where WNV occurs, USUV may also circulate (Roesch, Fajardo, Moratorio, & Vignuzzi, 2019). In another hand, as WNV and USUV E proteins share structural features, cross reactivity of antibodies may complicate the interpretation of results. The bird species identified as hosts of WNV in Iran also have been implicated in transmission cycles of Flaviviruses in Europe, including Spain (Jiménez‐Clavero et al., 2008), Slovakia (Csank et al., 2018), Germany (Michel et al., 2019) and Austria (Buchebner et al., 2013). Since the emergence of USUV in Europe in 1996, this virus has caused high numbers of bird deaths (Weissenböck, Bakonyi, Rossi, Mani, & Nowotny, 2013). USUV infections have been also detected in horses in Poland (Bażanów et al., 2018), Croatia (Barbic et al., 2013) and Italy (Savini et al., 2011), suggesting an active circulation of USUV in Europe with sporadic human cases in Hungary (Nagy et al., 2019), Italy (Percivalle et al., 2017), Germany (Cadar et al., 2017), France and Croatia (Santini et al., 2015).

Information obtained in this investigation highlights the need for maintaining active surveillance of WNV and USUV in their vertebrate hosts, as well as in mosquito vectors. This will help keeping track of their geographic spread and implement the appropriate measures.

## CONFLICT OF INTEREST

We have no competing interests.

## AUTHOR CONTRIBUTION

Hasan Bakhshi: Conceptualization; Methodology; Investigation; Writing‐original draft; Writing‐review & editing. Cécile Beck: Investigation; Methodology; Validation; Visualization; Writing‐review & editing. Sylvie Lecollinet: Investigation; Methodology; Validation; Visualization; Writing‐review & editing. Maëlle Monier: Validation; Visualization. Laurence Mousson: Data curation; Investigation; Methodology. Sedigheh Zakeri: Conceptualization; Methodology; Investigation; Supervision. Abbasali Raz: Conceptualization; Methodology; Investigation; Supervision. Kourosh Arzamani: Investigation. Leila Nourani: Investigation. Navid Dinparast‐Djadid: Conceptualization; Project administration; Methodology; Investigation; Funding acquisition; Supervision; Visualization; Writing‐review & editing. Anna‐Bella Failloux: Conceptualization; Formal analysis; Funding acquisition; Project administration; Supervision; Validation; Visualization; Writing‐review & editing.

### PEER REVIEW

The peer review history for this article is available at https://publons.com/publon/10.1002/vms3.342.

## Supporting information

Table S1

Table S2

Table S3

Table S4

## Data Availability

The data that support the findings of this study are available from the co‐corresponding authors upon reasonable request.
