# Supplementary material for: Serological evidence of West Nile virus infection among birds and horses in some geographical locations of Iran
Source: Vet Med Sci. 2020 Aug 28;7(1):204–9. doi: 10.1002/vms3.342 (PMC7840194; doi:10.1002/vms3.342)
Supplement: Supplementary file 1 — Table S1 [file VMS3-7-204-s001.docx]

**Table S1.** Details of bird species, their collection sites and gender/age of horse samples

| ***Genus species*** | **Place of collection** | **Gender** | **Age** |
| --- | --- | --- | --- |
| *Accipiter nisus* | Amol, Amol County, Mazandaran Province | NA | NA |
| *Alectoris chukar* | Sanandaj, Sanandaj County, Kordestan Province | NA | NA |
| *Alectoris chukar* | Sanandaj, Sanandaj County, Kordestan Province | NA | NA |
| *Alectoris chukar* | Sanandaj, Sanandaj County, Kordestan Province | NA | NA |
| *Alectoris chukar* | Sanandaj, Sanandaj County, Kordestan Province | NA | NA |
| *Alectoris chukar* | Sanandaj, Sanandaj County, Kordestan Province | NA | NA |
| *Anas acuta* | Ezbaran, Fereydunkenar County, Mazandaran Province | NA | NA |
| *Anas acuta* | Ezbaran, Fereydunkenar County, Mazandaran Province | NA | NA |
| *Anas acuta* | Ezbaran, Fereydunkenar County, Mazandaran Province | NA | NA |
| *Anas acuta* | Ezbaran, Fereydunkenar County, Mazandaran Province | NA | NA |
| *Anas crecca* | Ezbaran, Fereydunkenar County, Mazandaran Province | NA | NA |
| *Anas crecca* | Fereydunkenar, Fereydunkenar County, Mazandaran Province | NA | NA |
| *Anas crecca* | Sorkhrud, Mahmudabad County, Mazandaran Province | NA | NA |
| *Anas crecca* | Sorkhrud, Mahmudabad County, Mazandaran Province | NA | NA |
| *Anas crecca* | Ezbaran, Fereydunkenar County, Mazandaran Province | NA | NA |
| *Anas crecca* | Ezbaran, Fereydunkenar County, Mazandaran Province | NA | NA |
| *Anas crecca* | Ezbaran, Fereydunkenar County, Mazandaran Province | NA | NA |
| *Anas crecca* | Babol, Babol County, Mazandaran Province | NA | NA |
| *Anas crecca* | Fereydunkenar, Fereydunkenar County, Mazandaran Province | NA | NA |
| *Anas crecca* | Babol, Babol County, Mazandaran Province | NA | NA |
| *Anas crecca* | Babol, Babol County, Mazandaran Province | NA | NA |
| *Anas crecca* | Babol, Babol County, Mazandaran Province | NA | NA |
| *Anas crecca* | Fereydunkenar, Fereydunkenar County, Mazandaran Province | NA | NA |
| *Anas crecca* | Ezbaran, Fereydunkenar County, Mazandaran Province | NA | NA |
| *Anas crecca* | Babol, Babol County, Mazandaran Province | NA | NA |
| *Anas crecca* | Fereydunkenar, Fereydunkenar County, Mazandaran Province | NA | NA |
| *Anas crecca* | Babol, Babol County, Mazandaran Province | NA | NA |
| *Anas crecca* | Fereydunkenar, Fereydunkenar County, Mazandaran Province | NA | NA |
| *Anas crecca* | Babol, Babol County, Mazandaran Province | NA | NA |
| *Anas crecca* | Fereydunkenar, Fereydunkenar County, Mazandaran Province | NA | NA |
| *Anas crecca* | Fereydunkenar, Fereydunkenar County, Mazandaran Province | NA | NA |
| *Anas crecca* | Ezbaran, Fereydunkenar County, Mazandaran Province | NA | NA |
| *Anas crecca* | Babol, Babol County, Mazandaran Province | NA | NA |
| *Anas crecca* | Ezbaran, Fereydunkenar County, Mazandaran Province | NA | NA |
| *Anas crecca* | Fereydunkenar, Fereydunkenar County, Mazandaran Province | NA | NA |
| *Anas crecca* | Ezbaran, Fereydunkenar County, Mazandaran Province | NA | NA |
| *Anas crecca* | Fereydunkenar, Fereydunkenar County, Mazandaran Province | NA | NA |
| *Anas crecca* | Ezbaran, Fereydunkenar County, Mazandaran Province | NA | NA |
| *Anas crecca* | Babol, Babol County, Mazandaran Province | NA | NA |
| *Anas crecca* | Babol, Babol County, Mazandaran Province | NA | NA |
| *Anas crecca* | Ezbaran, Fereydunkenar County, Mazandaran Province | NA | NA |
| *Anas crecca* | Fereydunkenar, Fereydunkenar County, Mazandaran Province | NA | NA |
| *Anas crecca* | Ezbaran, Fereydunkenar County, Mazandaran Province | NA | NA |
| *Anas crecca* | Ezbaran, Fereydunkenar County, Mazandaran Province | NA | NA |
| *Anas crecca* | Babol, Babol County, Mazandaran Province | NA | NA |
| *Anas crecca* | Fereydunkenar, Fereydunkenar County, Mazandaran Province | NA | NA |
| *Anas crecca* | Ezbaran, Fereydunkenar County, Mazandaran Province | NA | NA |
| *Anas crecca* | Fereydunkenar, Fereydunkenar County, Mazandaran Province | NA | NA |
| *Anas crecca* | Babol, Babol County, Mazandaran Province | NA | NA |
| *Anas crecca* | Ezbaran, Fereydunkenar County, Mazandaran Province | NA | NA |
| *Anas crecca* | Fereydunkenar, Fereydunkenar County, Mazandaran Province | NA | NA |
| *Anas crecca* | Fereydunkenar, Fereydunkenar County, Mazandaran Province | NA | NA |
| *Anas crecca* | Fereydunkenar, Fereydunkenar County, Mazandaran Province | NA | NA |
| *Anas crecca* | Babol, Babol County, Mazandaran Province | NA | NA |
| *Anas crecca* | Fereydunkenar, Fereydunkenar County, Mazandaran Province | NA | NA |
| *Anas crecca* | Fereydunkenar, Fereydunkenar County, Mazandaran Province | NA | NA |
| *Anas crecca* | Babol, Babol County, Mazandaran Province | NA | NA |
| *Anas crecca* | Babol, Babol County, Mazandaran Province | NA | NA |
| *Anas crecca* | Amol, Amol County, Mazandaran Province | NA | NA |
| *Anas platyrhynchos* | Sorkhrud, Mahmudabad County, Mazandaran Province | NA | NA |
| *Anas platyrhynchos* | Babol, Babol County, Mazandaran Province | NA | NA |
| *Anas platyrhynchos* | Ezbaran, Fereydunkenar County, Mazandaran Province | NA | NA |
| *Anas platyrhynchos* | Babol, Babol County, Mazandaran Province | NA | NA |
| *Anas platyrhynchos* | Fereydunkenar, Fereydunkenar County, Mazandaran Province | NA | NA |
| *Anas platyrhynchos* | Babol, Babol County, Mazandaran Province | NA | NA |
| *Anas platyrhynchos* | Ezbaran, Fereydunkenar County, Mazandaran Province | NA | NA |
| *Anas platyrhynchos* | Fereydunkenar, Fereydunkenar County, Mazandaran Province | NA | NA |
| *Aquila chrysaetos* | Amol, Amol County, Mazandaran Province | NA | NA |
| *Aquila chrysaetos* | Amol, Amol County, Mazandaran Province | NA | NA |
| *Aquila chrysaetos* | Fereydunkenar, Fereydunkenar County, Mazandaran Province | NA | NA |
| *Aquila chrysaetos* | Savadkouh, Savadkouh County, Mazandaran Province | NA | NA |
| *Aquila chrysaetos* | Bojnurd, Bojnurd County, North Khorasan Province | NA | NA |
| *Aquila chrysaetos* | Bojnurd, Bojnurd County, North Khorasan Province | NA | NA |
| *Aquila chrysaetos* | Bojnurd, Bojnurd County, North Khorasan Province | NA | NA |
| *Aquila chrysaetos* | Bojnurd, Bojnurd County, North Khorasan Province | NA | NA |
| *Aquila chrysaetos* | Bojnurd, Bojnurd County, North Khorasan Province | NA | NA |
| *Aquila clanga* | Genareh, Gorgan County, Golestan Province | NA | NA |
| *Aquila clanga* | Genareh, Gorgan County, Golestan Province | NA | NA |
| *Aquila clanga* | Genareh, Gorgan County, Golestan Province | NA | NA |
| *Aquila clanga* | Genareh, Gorgan County, Golestan Province | NA | NA |
| *Aquila clanga* | Genareh, Gorgan County, Golestan Province | NA | NA |
| *Aquila heliaca* | Amol, Amol County, Mazandaran Province | NA | NA |
| *Aquila heliaca* | Amol, Amol County, Mazandaran Province | NA | NA |
| *Aquila pomarina* | Genareh, Gorgan County, Golestan Province | NA | NA |
| *Aquila rapax* | Genareh, Gorgan County, Golestan Province | NA | NA |
| *Aquila sp.* | Sanandaj, Sanandaj County, Kordestan Province | NA | NA |
| *Aquila sp.* | Genareh, Gorgan County, Golestan Province | NA | NA |
| *Asio flammeus* | Amol, Amol County, Mazandaran Province | NA | NA |
| *Aythya ferina* | Babol, Babol County, Mazandaran Province | NA | NA |
| *Aythya ferina* | Babol, Babol County, Mazandaran Province | NA | NA |
| *Aythya ferina* | Babol, Babol County, Mazandaran Province | NA | NA |
| *Bubo bubo* | Genareh, Gorgan County, Golestan Province | NA | NA |
| *Bubo bubo* | Bojnurd, Bojnurd County, North Khorasan Province | NA | NA |
| *Buteo buteo* | Amol, Amol County, Mazandaran Province | NA | NA |
| *Buteo buteo* | Amol, Amol County, Mazandaran Province | NA | NA |
| *Buteo buteo* | Bojnurd, Bojnurd County, North Khorasan Province | NA | NA |
| *Buteo buteo* | Bojnurd, Bojnurd County, North Khorasan Province | NA | NA |
| *Buteo buteo* | Bojnurd, Bojnurd County, North Khorasan Province | NA | NA |
| *Buteo rufinus* | Amol, Amol County, Mazandaran Province | NA | NA |
| *Buteo rufinus* | Genareh, Gorgan County, Golestan Province | NA | NA |
| *Buteo rufinus* | Genareh, Gorgan County, Golestan Province | NA | NA |
| *Buteo rufinus* | Genareh, Gorgan County, Golestan Province | NA | NA |
| *Buteo rufinus* | Genareh, Gorgan County, Golestan Province | NA | NA |
| *Buteo rufinus* | Genareh, Gorgan County, Golestan Province | NA | NA |
| *Buteo rufinus* | Genareh, Gorgan County, Golestan Province | NA | NA |
| *Buteo rufinus* | Genareh, Gorgan County, Golestan Province | NA | NA |
| *Buteo rufinus* | Genareh, Gorgan County, Golestan Province | NA | NA |
| *Buteo rufinus* | Genareh, Gorgan County, Golestan Province | NA | NA |
| *Buteo rufinus* | Genareh, Gorgan County, Golestan Province | NA | NA |
| *Circus cyaneus* | Amol, Amol County, Mazandaran Province | NA | NA |
| *Circus cyaneus* | Fereydunkenar, Fereydunkenar County, Mazandaran Province | NA | NA |
| *Circus cyaneus* | Babol, Babol County, Mazandaran Province | NA | NA |
| *Columba livia* | Sanandaj, Sanandaj County, Kordestan Province | NA | NA |
| *Columba livia* | Sanandaj, Sanandaj County, Kordestan Province | NA | NA |
| *Corvus monedula* | Bojnurd, Bojnurd County, North Khorasan Province | NA | NA |
| *Coturnix coturnix* | Sanandaj, Sanandaj County, Kordestan Province | NA | NA |
| *Coturnix coturnix* | Sanandaj, Sanandaj County, Kordestan Province | NA | NA |
| *Coturnix coturnix* | Sanandaj, Sanandaj County, Kordestan Province | NA | NA |
| *Coturnix coturnix* | Sanandaj, Sanandaj County, Kordestan Province | NA | NA |
| *Coturnix coturnix* | Sanandaj, Sanandaj County, Kordestan Province | NA | NA |
| *Coturnix coturnix* | Sanandaj, Sanandaj County, Kordestan Province | NA | NA |
| *Coturnix coturnix* | Sanandaj, Sanandaj County, Kordestan Province | NA | NA |
| *Coturnix coturnix* | Sanandaj, Sanandaj County, Kordestan Province | NA | NA |
| *Coturnix coturnix* | Sanandaj, Sanandaj County, Kordestan Province | NA | NA |
| *Coturnix coturnix* | Sanandaj, Sanandaj County, Kordestan Province | NA | NA |
| *Coturnix coturnix* | Sanandaj, Sanandaj County, Kordestan Province | NA | NA |
| *Equus ferus caballus* | Gonbad-Kavus, Gonbad-Kavus County, Golestan Province | M | 3 |
| *Equus ferus caballus* | Gonbad-Kavus, Gonbad-Kavus County, Golestan Province | F | 2 |
| *Equus ferus caballus* | Gonbad-Kavus, Gonbad-Kavus County, Golestan Province | M | 2 |
| *Equus ferus caballus* | Gonbad-Kavus, Gonbad-Kavus County, Golestan Province | M | 3 |
| *Equus ferus caballus* | Gonbad-Kavus, Gonbad-Kavus County, Golestan Province | M | 2 |
| *Equus ferus caballus* | Gonbad-Kavus, Gonbad-Kavus County, Golestan Province | F | 3 |
| *Equus ferus caballus* | Gonbad-Kavus, Gonbad-Kavus County, Golestan Province | F | 2 |
| *Equus ferus caballus* | Gonbad-Kavus, Gonbad-Kavus County, Golestan Province | F | 3 |
| *Equus ferus caballus* | Gonbad-Kavus, Gonbad-Kavus County, Golestan Province | M | 2 |
| *Equus ferus caballus* | Gonbad-Kavus, Gonbad-Kavus County, Golestan Province | F | 2 |
| *Equus ferus caballus* | Gonbad-Kavus, Gonbad-Kavus County, Golestan Province | M | 2 |
| *Equus ferus caballus* | Gonbad-Kavus, Gonbad-Kavus County, Golestan Province | M | 2 |
| *Equus ferus caballus* | Gonbad-Kavus, Gonbad-Kavus County, Golestan Province | M | 3 |
| *Equus ferus caballus* | Gonbad-Kavus, Gonbad-Kavus County, Golestan Province | M | 2 |
| *Equus ferus caballus* | Gonbad-Kavus, Gonbad-Kavus County, Golestan Province | M | 2 |
| *Equus ferus caballus* | Gonbad-Kavus, Gonbad-Kavus County, Golestan Province | F | 2 |
| *Equus ferus caballus* | Gonbad-Kavus, Gonbad-Kavus County, Golestan Province | M | 2 |
| *Equus ferus caballus* | Gonbad-Kavus, Gonbad-Kavus County, Golestan Province | M | 2 |
| *Equus ferus caballus* | Gonbad-Kavus, Gonbad-Kavus County, Golestan Province | F | 2 |
| *Equus ferus caballus* | Gonbad-Kavus, Gonbad-Kavus County, Golestan Province | F | 2 |
| *Equus ferus caballus* | Gonbad-Kavus, Gonbad-Kavus County, Golestan Province | F | 1.5 |
| *Equus ferus caballus* | Gonbad-Kavus, Gonbad-Kavus County, Golestan Province | F | 1.5 |
| *Equus ferus caballus* | Gonbad-Kavus, Gonbad-Kavus County, Golestan Province | M | 2 |
| *Equus ferus caballus* | Gonbad-Kavus, Gonbad-Kavus County, Golestan Province | F | 2 |
| *Equus ferus caballus* | Gonbad-Kavus, Gonbad-Kavus County, Golestan Province | F | 3 |
| *Equus ferus caballus* | Gonbad-Kavus, Gonbad-Kavus County, Golestan Province | M | 5 |
| *Equus ferus caballus* | Gonbad-Kavus, Gonbad-Kavus County, Golestan Province | F | 2 |
| *Equus ferus caballus* | Gonbad-Kavus, Gonbad-Kavus County, Golestan Province | F | 3 |
| *Equus ferus caballus* | Gonbad-Kavus, Gonbad-Kavus County, Golestan Province | F | 1.5 |
| *Equus ferus caballus* | Gonbad-Kavus, Gonbad-Kavus County, Golestan Province | M | 3 |
| *Equus ferus caballus* | Gonbad-Kavus, Gonbad-Kavus County, Golestan Province | F | 4 |
| *Equus ferus caballus* | Gonbad-Kavus, Gonbad-Kavus County, Golestan Province | M | 3 |
| *Equus ferus caballus* | Gonbad-Kavus, Gonbad-Kavus County, Golestan Province | M | 2 |
| *Equus ferus caballus* | Gonbad-Kavus, Gonbad-Kavus County, Golestan Province | M | 2 |
| *Equus ferus caballus* | Gonbad-Kavus, Gonbad-Kavus County, Golestan Province | M | 1.5 |
| *Equus ferus caballus* | Gonbad-Kavus, Gonbad-Kavus County, Golestan Province | M | 2 |
| *Equus ferus caballus* | Gonbad-Kavus, Gonbad-Kavus County, Golestan Province | M | 3 |
| *Equus ferus caballus* | Gonbad-Kavus, Gonbad-Kavus County, Golestan Province | M | 3 |
| *Equus ferus caballus* | Gonbad-Kavus, Gonbad-Kavus County, Golestan Province | M | 1.5 |
| *Equus ferus caballus* | Gonbad-Kavus, Gonbad-Kavus County, Golestan Province | M | 2 |
| *Equus ferus caballus* | Gonbad-Kavus, Gonbad-Kavus County, Golestan Province | F | 2 |
| *Equus ferus caballus* | Gonbad-Kavus, Gonbad-Kavus County, Golestan Province | F | 3 |
| *Equus ferus caballus* | Gonbad-Kavus, Gonbad-Kavus County, Golestan Province | F | 3 |
| *Equus ferus caballus* | Gonbad-Kavus, Gonbad-Kavus County, Golestan Province | M | 2 |
| *Equus ferus caballus* | Gonbad-Kavus, Gonbad-Kavus County, Golestan Province | M | 2 |
| *Equus ferus caballus* | Gonbad-Kavus, Gonbad-Kavus County, Golestan Province | M | 1.5 |
| *Equus ferus caballus* | Gonbad-Kavus, Gonbad-Kavus County, Golestan Province | M | 1 |
| *Equus ferus caballus* | Gonbad-Kavus, Gonbad-Kavus County, Golestan Province | M | 5 |
| *Equus ferus caballus* | Gonbad-Kavus, Gonbad-Kavus County, Golestan Province | M | 2 |
| *Equus ferus caballus* | Gonbad-Kavus, Gonbad-Kavus County, Golestan Province | F | 2 |
| *Equus ferus caballus* | Gonbad-Kavus, Gonbad-Kavus County, Golestan Province | F | 3 |
| *Equus ferus caballus* | Gonbad-Kavus, Gonbad-Kavus County, Golestan Province | M | 6 |
| *Equus ferus caballus* | Gonbad-Kavus, Gonbad-Kavus County, Golestan Province | M | 2 |
| *Equus ferus caballus* | Gonbad-Kavus, Gonbad-Kavus County, Golestan Province | F | 2 |
| *Equus ferus caballus* | Gonbad-Kavus, Gonbad-Kavus County, Golestan Province | M | 3 |
| *Equus ferus caballus* | Gonbad-Kavus, Gonbad-Kavus County, Golestan Province | M | 2 |
| *Equus ferus caballus* | Gonbad-Kavus, Gonbad-Kavus County, Golestan Province | M | 2 |
| *Equus ferus caballus* | Gonbad-Kavus, Gonbad-Kavus County, Golestan Province | F | 2.5 |
| *Equus ferus caballus* | Gonbad-Kavus, Gonbad-Kavus County, Golestan Province | F | 2 |
| *Equus ferus caballus* | Gonbad-Kavus, Gonbad-Kavus County, Golestan Province | M | 3 |
| *Falco naumanni* | Genareh, Gorgan County, Golestan Province | NA | NA |
| *Falco tinnunculus* | Amol, Amol County, Mazandaran Province | NA | NA |
| *Falco tinnunculus* | Behshahr, Behshahr County, Mazandaran Province | NA | NA |
| *Falco tinnunculus* | Genareh, Gorgan County, Golestan Province | NA | NA |
| *Falco tinnunculus* | Genareh, Gorgan County, Golestan Province | NA | NA |
| *Fulica atra* | Ezbaran, Fereydunkenar County, Mazandaran Province | NA | NA |
| *Fulica atra* | Ezbaran, Fereydunkenar County, Mazandaran Province | NA | NA |
| *Fulica atra* | Ezbaran, Fereydunkenar County, Mazandaran Province | NA | NA |
| *Fulica atra* | Ezbaran, Fereydunkenar County, Mazandaran Province | NA | NA |
| *Fulica atra* | Babol, Babol County, Mazandaran Province | NA | NA |
| *Fulica atra* | Babol, Babol County, Mazandaran Province | NA | NA |
| *Fulica atra* | Babol, Babol County, Mazandaran Province | NA | NA |
| *Fulica atra* | Babol, Babol County, Mazandaran Province | NA | NA |
| *Fulica atra* | Babol, Babol County, Mazandaran Province | NA | NA |
| *Fulica atra* | Babol, Babol County, Mazandaran Province | NA | NA |
| *Fulica atra* | Babol, Babol County, Mazandaran Province | NA | NA |
| *Fulica atra* | Babol, Babol County, Mazandaran Province | NA | NA |
| *Fulica atra* | Ezbaran, Fereydunkenar County, Mazandaran Province | NA | NA |
| *Fulica atra* | Babol, Babol County, Mazandaran Province | NA | NA |
| *Fulica atra* | Babol, Babol County, Mazandaran Province | NA | NA |
| *Gallus gallus* | Sanandaj, Sanandaj County, Kordestan Province | NA | NA |
| *Gallus gallus* | Sanandaj, Sanandaj County, Kordestan Province | NA | NA |
| *Gallus gallus* | Sanandaj, Sanandaj County, Kordestan Province | NA | NA |
| *Haliaeetus albicilla* | Genareh, Gorgan County, Golestan Province | NA | NA |
| *Hydrocoloeus minutus* | Babol, Babol County, Mazandaran Province | NA | NA |
| *Meleagris gallopavo* | Sanandaj, Sanandaj County, Kordestan Province | NA | NA |
| *Meleagris gallopavo* | Sanandaj, Sanandaj County, Kordestan Province | NA | NA |
| *Milvus migrans* | Genareh, Gorgan County, Golestan Province | NA | NA |
| *Milvus migrans* | Genareh, Gorgan County, Golestan Province | NA | NA |
| *Neophron percnopterus* | Genareh, Gorgan County, Golestan Province | NA | NA |
| *Neophron percnopterus* | Genareh, Gorgan County, Golestan Province | NA | NA |
| *Strix aluco* | Amol, Amol County, Mazandaran Province | NA | NA |
| *Strix aluco* | Behshahr, Behshahr County, Mazandaran Province | NA | NA |
| *Strix aluco* | Sari, Sari County, Mazandaran Province | NA | NA |
