# Supplementary material for: Serological evidence of West Nile virus infection among birds and horses in some geographical locations of Iran
Source: Vet Med Sci. 2020 Aug 28;7(1):204–9. doi: 10.1002/vms3.342 (PMC7840194; doi:10.1002/vms3.342)
Supplement: Supplementary file 2 — Table S2 [file VMS3-7-204-s002.docx]

**Table S2.** Details of blood samples collected in four provinces: Mazandaran (Amol, Amol County: 13; Ezbaran, Fereydunkenar County: 25; Fereydunkenar, Fereydunkenar County: 21; Sorkhrud, Mahmudabad County: 3; Babol, Babol County: 33; Behshahr, Behshahr County: 2; Sari, Sari county: 1; Savadkouh, Savadkouh, county: 1), Kordestan (Sanandaj, Sanandaj County: 24), Golestan (Genareh, Gorgan County: 27), and North Khorasan Province (Bojnurd, Bojnurd County: 10). A total of 220 bird and horse blood samples were collected.

| **Host** | **Family** | **Genus** | **Common name** | **Scientific name** | **No. of samples collected** | **No. of positive samples** |
| --- | --- | --- | --- | --- | --- | --- |
| **Birds** | Accipitridae | *Accipiter* | Eurasian sparrowhawk | *Accipiter nisus* | 1 |  |
|  |  | *Aquila* | Golden eagle | *Aquila chrysaetos* | 9 | 1 |
|  |  |  | Greater spotted eagle | *Aquila clanga* | 5 |  |
|  |  |  | Eastern imperial eagle | *Aquila heliaca* | 2 |  |
|  |  |  | Lesser spotted eagle | *Aquila pomarina* | 1 |  |
|  |  |  | Tawny eagle | *Aquila rapax* | 1 |  |
|  |  |  | NA | *Aquila sp.* | 2 |  |
|  |  | *Buteo* | Common buzzard | *Buteo buteo* | 5 |  |
|  |  |  | Long-legged buzzard | *Buteo rufinus* | 11 | 1 |
|  |  | *Circus* | Hen harrier | *Circus cyaneus* | 3 |  |
|  |  | *Haliaeetus* | White-tailed eagle | *Haliaeetus albicilla* | 1 |  |
|  |  | *Milvus* | Black kite | *Milvus migrans* | 2 |  |
|  |  | *Neophron* | Egyptian vulture | *Neophron percnopterus* | 2 | 2 |
|  | Anatidae | *Anas* | Northern pintail | *Anas acuta* | 4 |  |
|  |  |  | Eurasian teal | *Anas crecca* | 49 |  |
|  |  |  | Mallard | *Anas platyrhynchos* | 8 |  |
|  |  | *Aythya* | Common pochard | *Aythya ferina* | 3 |  |
|  | Columbidae | *Columba* | Rock dove | *Columba livia* | 2 |  |
|  | Corvidae | *Corvus* | Western jackdaw | *Corvus monedula* | 1 |  |
|  | Falconidae | *Falco* | Lesser kestrel | *Falco naumanni* | 1 |  |
|  |  |  | Common kestrel | *Falco tinnunculus* | 4 |  |
|  | Laridae | *Hydrocoloeus* | Little gull | *Hydrocoloeus minutus* | 1 |  |
|  | Phasianidae | *Alectoris* | Chukar partridge | *Alectoris chukar* | 5 |  |
|  |  | *Coturnix* | Common quail | *Coturnix coturnix* | 11 |  |
|  |  | *Gallus* | Red junglefowl | *Gallus gallus* | 3 |  |
|  |  | *Meleagris* | Wild turkey | *Meleagris gallopavo* | 2 |  |
|  | Rallidae | *Fulica* | Common coot | *Fulica atra* | 15 |  |
|  | Strigidae | *Asio* | Short-eared owl | *Asio flammeus* | 1 |  |
|  |  | *Bubo* | Eurasian eagle-owl | *Bubo bubo* | 2 |  |
|  |  | *Strix* | Tawny owl | *Strix aluco* | 3 |  |
| **Horses** | Equidae | *Equus* | Horse | *Equus ferus caballus* | 60 | 10 |
| **Total** | **10** | **22** | **30** | **30** | **220** | **14** |
