# Supplementary material for: Serological evidence of West Nile virus infection among birds and horses in some geographical locations of Iran
Source: Vet Med Sci. 2020 Aug 28;7(1):204–9. doi: 10.1002/vms3.342 (PMC7840194; doi:10.1002/vms3.342)
Supplement: Supplementary file 3 — Table S3 [file VMS3-7-204-s003.docx]

**Table S3.** Details of samples positive with the ID Screen WNV competition ELISA (ID ScreenWest Nile Competition; ID VET, France; according to the manufacturer’s instructions). Out of 220 collected samples, 32 samples (14.54%) including 22 birds and 10 horses, were positive (positive: %S/N < 40%; doubtful: 40% <S/N<50%; negative: S/N>50%). Samples are placed according to the place of collection, the %S/N and in alphabetic order.

| positive: | %S/N < 40% |
| --- | --- |
| doubtful: | 40% <S/N<50% |
| negative: | S/N>50% |

| **Species** | **Place of collection** | **% S/N** |
| --- | --- | --- |
| *Buteo rufinus* | Golestan | 9.5 |
| *Buteo rufinus* | Golestan | 8.7 |
| *Falco tinnunculus* | Golestan | 10.2 |
| *Neophron percnopterus* | Golestan | 5.2 |
| *Neophron percnopterus* | Golestan | 32.7 |
| *Aquila clanga* | Golestan | 65.3 |
| *Aquila clanga* | Golestan | 84.5 |
| *Aquila clanga* | Golestan | 75.5 |
| *Aquila clanga* | Golestan | 84.5 |
| *Aquila clanga* | Golestan | 83.6 |
| *Aquila pomarina* | Golestan | 88.0 |
| *Aquila rapax* | Golestan | 78.2 |
| *Aquila sp.* | Golestan | 85.7 |
| *Bubo bubo* | Golestan | 81.6 |
| *Buteo rufinus* | Golestan | 83.5 |
| *Buteo rufinus* | Golestan | 84.6 |
| *Buteo rufinus* | Golestan | 84.3 |
| *Buteo rufinus* | Golestan | 59.4 |
| *Buteo rufinus* | Golestan | 84.5 |
| *Buteo rufinus* | Golestan | 84.8 |
| *Buteo rufinus* | Golestan | 83.6 |
| *Buteo rufinus* | Golestan | 88.4 |
| *Falco naumanni* | Golestan | 82.2 |
| *Falco tinnunculus* | Golestan | 80.5 |
| *Haliaeetus albicilla* | Golestan | 88.0 |
| *Hydrocoloeus minutus* | Mazandaran | 94.0 |
| *Milvus migrans* | Golestan | 84.2 |
| *Milvus migrans* | Golestan | 80.8 |
|  | | |
| *Anas crecca* | Mazandaran | 26.0 |
| *Anas crecca* | Mazandaran | 4.9 |
| *Aquila chrysaetos* | Mazandaran | 8.4 |
| *Aquila heliaca* | Mazandaran | 39.4 |
| *Buteo buteo* | Mazandaran | 23.4 |
| *Fulica atra* | Mazandaran | 3.7 |
| *Fulica atra* | Mazandaran | 5.2 |
| *Fulica atra* | Mazandaran | 5.9 |
| *Fulica atra* | Mazandaran | 6.3 |
| *Fulica atra* | Mazandaran | 8.4 |
| *Fulica atra* | Mazandaran | 8.8 |
| *Fulica atra* | Mazandaran | 15.5 |
| *Fulica atra* | Mazandaran | 15.8 |
| *Fulica atra* | Mazandaran | 17.5 |
| *Fulica atra* | Mazandaran | 37.4 |
| *Aquila chrysaetos* | Mazandaran | 49.6 |
| *Fulica atra* | Mazandaran | 41.2 |
| *Accipiter nisus* | Mazandaran | 67.2 |
| *Anas acuta* | Mazandaran | 80 |
| *Anas acuta* | Mazandaran | 80.9 |
| *Anas acuta* | Mazandaran | 81.9 |
| *Anas acuta* | Mazandaran | 82.0 |
| *Anas crecca* | Mazandaran | 70.8 |
| *Anas crecca* | Mazandaran | 71.2 |
| *Anas crecca* | Mazandaran | 72.0 |
| *Anas crecca* | Mazandaran | 73.9 |
| *Anas crecca* | Mazandaran | 73.9 |
| *Anas crecca* | Mazandaran | 74.4 |
| *Anas crecca* | Mazandaran | 74.7 |
| *Anas crecca* | Mazandaran | 76.3 |
| *Anas crecca* | Mazandaran | 76.3 |
| *Anas crecca* | Mazandaran | 76.3 |
| *Anas crecca* | Mazandaran | 77.0 |
| *Anas crecca* | Mazandaran | 77.0 |
| *Anas crecca* | Mazandaran | 77.7 |
| *Anas crecca* | Mazandaran | 78.0 |
| *Anas crecca* | Mazandaran | 78.2 |
| *Anas crecca* | Mazandaran | 78.3 |
| *Anas crecca* | Mazandaran | 79.7 |
| *Anas crecca* | Mazandaran | 79.7 |
| *Anas crecca* | Mazandaran | 79.9 |
| *Anas crecca* | Mazandaran | 80.3 |
| *Anas crecca* | Mazandaran | 80.7 |
| *Anas crecca* | Mazandaran | 80.8 |
| *Anas crecca* | Mazandaran | 81.4 |
| *Anas crecca* | Mazandaran | 81.7 |
| *Anas crecca* | Mazandaran | 82.5 |
| *Anas crecca* | Mazandaran | 83.0 |
| *Anas crecca* | Mazandaran | 83.1 |
| *Anas crecca* | Mazandaran | 83.2 |
| *Anas crecca* | Mazandaran | 83.8 |
| *Anas crecca* | Mazandaran | 83.9 |
| *Anas crecca* | Mazandaran | 84.9 |
| *Anas crecca* | Mazandaran | 85.2 |
| *Anas crecca* | Mazandaran | 85.4 |
| *Anas crecca* | Mazandaran | 85.7 |
| *Anas crecca* | Mazandaran | 86.6 |
| *Anas crecca* | Mazandaran | 86.7 |
| *Anas crecca* | Mazandaran | 86.7 |
| *Anas crecca* | Mazandaran | 87.0 |
| *Anas crecca* | Mazandaran | 87.5 |
| *Anas crecca* | Mazandaran | 88.1 |
| *Anas crecca* | Mazandaran | 89.2 |
| *Anas crecca* | Mazandaran | 89.8 |
| *Anas crecca* | Mazandaran | 89.9 |
| *Anas crecca* | Mazandaran | 96.1 |
| *Anas crecca* | Mazandaran | 99.2 |
| *Anas crecca* | Mazandaran | 102.5 |
| *Anas crecca* | Mazandaran | 102.8 |
| *Anas crecca* | Mazandaran | 106.9 |
| *Anas platyrhynchos* | Mazandaran | 75.9 |
| *Anas platyrhynchos* | Mazandaran | 79.7 |
| *Anas platyrhynchos* | Mazandaran | 80.5 |
| *Anas platyrhynchos* | Mazandaran | 81.4 |
| *Anas platyrhynchos* | Mazandaran | 82.6 |
| *Anas platyrhynchos* | Mazandaran | 85.9 |
| *Anas platyrhynchos* | Mazandaran | 86.6 |
| *Anas platyrhynchos* | Mazandaran | 96.7 |
| *Aquila chrysaetos* | Mazandaran | 86.5 |
| *Aquila chrysaetos* | Mazandaran | 106.7 |
| *Aquila heliaca* | Mazandaran | 63.2 |
| *Asio flammeus* | Mazandaran | 69.1 |
| *Aythya ferina* | Mazandaran | 71.1 |
| *Aythya ferina* | Mazandaran | 78.3 |
| *Aythya ferina* | Mazandaran | 93.6 |
| *Buteo buteo* | Mazandaran | 72.6 |
| *Buteo rufinus* | Mazandaran | 64.6 |
| *Circus cyaneus* | Mazandaran | 63.7 |
| *Circus cyaneus* | Mazandaran | 74.4 |
| *Circus cyaneus* | Mazandaran | 80.4 |
| *Falco tinnunculus* | Mazandaran | 77.6 |
| *Falco tinnunculus* | Mazandaran | 78.8 |
| *Strix aluco* | Mazandaran | 64.6 |
| *Fulica atra* | Mazandaran | 70.0 |
| *Fulica atra* | Mazandaran | 71.1 |
| *Fulica atra* | Mazandaran | 71.2 |
| *Strix aluco* | Mazandaran | 76.3 |
| *Fulica atra* | Mazandaran | 78.5 |
| *Strix aluco* | Mazandaran | 83.8 |
|  | | |
| *Columba livia* | Kordestan | 46.4 |
| *Alectoris chukar* | Kordestan | 81.6 |
| *Alectoris chukar* | Kordestan | 84.1 |
| *Alectoris chukar* | Kordestan | 85.0 |
| *Alectoris chukar* | Kordestan | 86.5 |
| *Alectoris chukar* | Kordestan | 86.8 |
| *Aquila sp.* | Kordestan | 83.4 |
| *Columba livia* | Kordestan | 72.9 |
| *Coturnix coturnix* | Kordestan | 63.1 |
| *Coturnix coturnix* | Kordestan | 64.2 |
| *Coturnix coturnix* | Kordestan | 71.8 |
| *Coturnix coturnix* | Kordestan | 72.8 |
| *Coturnix coturnix* | Kordestan | 73.8 |
| *Coturnix coturnix* | Kordestan | 74.7 |
| *Coturnix coturnix* | Kordestan | 75.6 |
| *Coturnix coturnix* | Kordestan | 78.5 |
| *Coturnix coturnix* | Kordestan | 79.8 |
| *Coturnix coturnix* | Kordestan | 83.0 |
| *Coturnix coturnix* | Kordestan | 83.7 |
| *Gallus gallus* | Kordestan | 79.6 |
| *Gallus gallus* | Kordestan | 80.4 |
| *Gallus gallus* | Kordestan | 81.5 |
| *Meleagris gallopavo* | Kordestan | 72.9 |
| *Meleagris gallopavo* | Kordestan | 73.9 |
|  | | |
| *Aquila chrysaetos* | North Khorasan | 4.5 |
| *Aquila chrysaetos* | North Khorasan | 10.3 |
| *Aquila chrysaetos* | North Khorasan | 77.6 |
| *Aquila chrysaetos* | North Khorasan | 82.3 |
| *Aquila chrysaetos* | North Khorasan | 83.7 |
| *Buteo buteo* | North Khorasan | 76.1 |
| *Buteo buteo* | North Khorasan | 78.6 |
| *Buteo buteo* | North Khorasan | 81.6 |
| *Bubo bubo* | North Khorasan | 82.0 |
| *Corvus monedula* | North Khorasan | 83.2 |
|  | | |
| *Equus ferus caballus* | Golestan | 4.4 |
| *Equus ferus caballus* | Golestan | 4.6 |
| *Equus ferus caballus* | Golestan | 4.7 |
| *Equus ferus caballus* | Golestan | 4.7 |
| *Equus ferus caballus* | Golestan | 4.7 |
| *Equus ferus caballus* | Golestan | 5.6 |
| *Equus ferus caballus* | Golestan | 6.5 |
| *Equus ferus caballus* | Golestan | 8.0 |
| *Equus ferus caballus* | Golestan | 9.1 |
| *Equus ferus caballus* | Golestan | 14.3 |
| *Equus ferus caballus* | Golestan | 66.7 |
| *Equus ferus caballus* | Golestan | 72.0 |
| *Equus ferus caballus* | Golestan | 76.1 |
| *Equus ferus caballus* | Golestan | 76.5 |
| *Equus ferus caballus* | Golestan | 76.7 |
| *Equus ferus caballus* | Golestan | 77.0 |
| *Equus ferus caballus* | Golestan | 77.4 |
| *Equus ferus caballus* | Golestan | 77.6 |
| *Equus ferus caballus* | Golestan | 78.3 |
| *Equus ferus caballus* | Golestan | 78.4 |
| *Equus ferus caballus* | Golestan | 78.8 |
| *Equus ferus caballus* | Golestan | 80.1 |
| *Equus ferus caballus* | Golestan | 80.4 |
| *Equus ferus caballus* | Golestan | 80.4 |
| *Equus ferus caballus* | Golestan | 80.6 |
| *Equus ferus caballus* | Golestan | 80.9 |
| *Equus ferus caballus* | Golestan | 81.0 |
| *Equus ferus caballus* | Golestan | 81.6 |
| *Equus ferus caballus* | Golestan | 81.8 |
| *Equus ferus caballus* | Golestan | 81.8 |
| *Equus ferus caballus* | Golestan | 82.2 |
| *Equus ferus caballus* | Golestan | 83.3 |
| *Equus ferus caballus* | Golestan | 83.7 |
| *Equus ferus caballus* | Golestan | 83.7 |
| *Equus ferus caballus* | Golestan | 83.7 |
| *Equus ferus caballus* | Golestan | 84.6 |
| *Equus ferus caballus* | Golestan | 86.4 |
| *Equus ferus caballus* | Golestan | 86.8 |
| *Equus ferus caballus* | Golestan | 87.9 |
| *Equus ferus caballus* | Golestan | 88.3 |
| *Equus ferus caballus* | Golestan | 88.8 |
| *Equus ferus caballus* | Golestan | 89.00 |
| *Equus ferus caballus* | Golestan | 89.4 |
| *Equus ferus caballus* | Golestan | 89.4 |
| *Equus ferus caballus* | Golestan | 89.71 |
| *Equus ferus caballus* | Golestan | 90.4 |
| *Equus ferus caballus* | Golestan | 90.5 |
| *Equus ferus caballus* | Golestan | 91.0 |
| *Equus ferus caballus* | Golestan | 91.5 |
| *Equus ferus caballus* | Golestan | 91.6 |
| *Equus ferus caballus* | Golestan | 91.9 |
| *Equus ferus caballus* | Golestan | 92.0 |
| *Equus ferus caballus* | Golestan | 92.4 |
| *Equus ferus caballus* | Golestan | 92.6 |
| *Equus ferus caballus* | Golestan | 93.2 |
| *Equus ferus caballus* | Golestan | 93.5 |
| *Equus ferus caballus* | Golestan | 93.54 |
| *Equus ferus caballus* | Golestan | 94.1 |
| *Equus ferus caballus* | Golestan | 97.3 |
| *Equus ferus caballus* | Golestan | >100 |
