# Supplementary material for: Serological evidence of West Nile virus infection among birds and horses in some geographical locations of Iran
Source: Vet Med Sci. 2020 Aug 28;7(1):204–9. doi: 10.1002/vms3.342 (PMC7840194; doi:10.1002/vms3.342)
Supplement: Supplementary file 4 — Table S4 [file VMS3-7-204-s004.docx]

**Table S4.** Results of WNV and USUV microneutralization tests on cELISA-birds positive samples. Microneutralization test results are expressed as negative or positive (i.e. titers ≥ 10) (* Two sera were cytotoxic and could not be evaluated by MNT).

| WNV  USUV | Negative | 40 | 160 | 320 | **Total** |
| --- | --- | --- | --- | --- | --- |
| Negative | 1 | 1 | 2 |  | **4** |
| 10 |  |  |  |  | **0** |
| 20 |  |  |  | 1 | **1** |
| **Total** | **1** | **1** | **2** | **1** | **5*** |
